# The microbiome associated with equine periodontitis and oral health

**DOI:** 10.1186/s13567-016-0333-1

**Published:** 2016-04-14

**Authors:** Rebekah Kennedy, David Francis Lappin, Padraic Martin Dixon, Mark Johannes Buijs, Egija Zaura, Wim Crielaard, Lindsay O’Donnell, David Bennett, Bernd Willem Brandt, Marcello Pasquale Riggio

**Affiliations:** Infection and Immunity Research Group, Dental School, University of Glasgow, 378 Sauchiehall Street, Glasgow, G2 3JZ UK; Division of Veterinary Clinical Studies, Royal (Dick) School of Veterinary Studies and Roslin Institute, University of Edinburgh, Easter Bush Veterinary Campus, Roslin, Midlothian, EH25 9RG UK; Department of Preventive Dentistry, Academic Centre for Dentistry Amsterdam, University of Amsterdam and VU University Amsterdam, Gustav Mahlerlaan 3004, 1081 LA Amsterdam, The Netherlands; School of Veterinary Medicine, University of Glasgow, 464 Bearsden Road, Glasgow, G61 1QH UK

## Abstract

Equine periodontal disease is a common and painful condition and its severe form, periodontitis, can lead to tooth loss. Its aetiopathogenesis remains poorly understood despite recent increased awareness of this disorder amongst the veterinary profession. Bacteria have been found to be causative agents of the disease in other species, but current understanding of their role in equine periodontitis is extremely limited. The aim of this study was to use high-throughput sequencing to identify the microbiome associated with equine periodontitis and oral health. Subgingival plaque samples from 24 horses with periodontitis and gingival swabs from 24 orally healthy horses were collected. DNA was extracted from samples, the V3–V4 region of the bacterial 16S rRNA gene amplified by PCR and amplicons sequenced using Illumina MiSeq. Data processing was conducted using USEARCH and QIIME. Diversity analyses were performed with PAST v3.02. Linear discriminant analysis effect size (LEfSe) was used to determine differences between the groups. In total, 1308 OTUs were identified and classified into 356 genera or higher taxa. Microbial profiles at health differed significantly from periodontitis, both in their composition (*p* < 0.0001, F = 12.24; PERMANOVA) and in microbial diversity (*p* < 0.001; Mann–Whitney test). Samples from healthy horses were less diverse (1.78, SD 0.74; Shannon diversity index) and were dominated by the genera *Gemella* and *Actinobacillus*, while the periodontitis group samples showed higher diversity (3.16, SD 0.98) and were dominated by the genera *Prevotella* and *Veillonella*. It is concluded that the microbiomes associated with equine oral health and periodontitis are distinct, with the latter displaying greater microbial diversity.

**Electronic supplementary material:**

The online version of this article (doi:10.1186/s13567-016-0333-1) contains supplementary material, which is available to authorized users.

## Introduction

Periodontal disease has long been recognised as a common and painful equine oral disorder and its substantial welfare impact was acknowledged at the start of the twentieth century being described as “the scourge of the horse’s mouth” [1, 2]. More recently, studies have shown the presence of periodontitis in up to 75% of horses [3, 4] with prevalence increasing with advancing age. A dental survey noted that classical (i.e. plaque-induced) periodontal disease was rare in horses, but periodontal disease induced by food impaction due to abnormal spacing between the cheek teeth was common [5]. The condition is often associated with the presence of cheek teeth diastemata [6] and can also be present secondary to other oral disorders such as supernumerary, displaced or rotated teeth [7]. Dropping of feed (quidding) and difficulty eating are the main clinical signs [8], although these can be subtle and easily overlooked. More recent clinical studies have reinforced the importance of equine periodontitis, currently recognised as a common and very painful equine dental disease [6, 8]. Two forms of periodontal disease exist, namely gingivitis and periodontitis. Gingivitis is completely reversible and is recognised by the classic signs of bleeding, inflammation, redness and swelling of the gums. Periodontitis attacks the deeper structures that support the teeth, damaging the surrounding bone and periodontal ligament, resulting in tooth loss. Despite the importance of this condition there have been few recent studies into its aetiopathogenesis.

Bacteria have been shown to be the causative agents in feline, canine and human periodontal disease and so it is highly likely they play a crucial role in the pathogenesis of the equine condition. Involvement of bacteria in equine periodontal disease was recently acknowledged [9, 10]. However, understanding of the equine oral microbiome is limited and merits further study and little is known about the role bacteria play in equine periodontitis [9]. Studies in other species have estimated that around 50% of oral bacteria cannot be cultured by conventional approaches due to nutritional and fastidious growth requirements [11] and thus the number and variety of bacterial species present in the oral microbiome has been greatly underestimated to date.

It is now possible to determine almost the entire community of bacteria, both commensal and pathogenic, that inhabit the equine oral cavity, in both health and periodontitis using culture-independent methods. To date, the majority of approaches have used Sanger sequencing to determine bacterial 16S rRNA gene sequences. This approach allows detection not only of cultivable species but also of fastidious bacteria that may be uncultivable, and also of novel species that may be important in the pathogenesis of disease. This method has already been used to determine the bacterial species present in canine [12] and ovine [13] periodontal disease lesions.

The aim of this study was to determine the microbial profiles associated with the healthy equine oral cavity and equine periodontitis using high-throughput sequencing of the bacterial 16S rRNA gene. This approach provides far greater depth, coverage, accuracy and sensitivity than that offered by Sanger sequencing in assessing the composition of complex microbial communities, uncovering microbial diversities that are orders of magnitude higher and with considerably less bias [14].

## Materials and methods

### Sample classification

Ethical approval was granted prior to the start of the study by the University of Glasgow School of Veterinary Medicine Ethics and Research Committee and by the University of Edinburgh Veterinary Ethical Review Committee. All horses involved in the study presented either to the Weipers Centre Equine Hospital, University of Glasgow or the Royal (Dick) School of Veterinary Studies, University of Edinburgh for routine dental examination, investigation of dental disease or had been humanely euthanatised for reasons unrelated to the oral cavity and sent for post-mortem examination. Following a thorough oral examination horses were categorised as either “orally healthy” or “periodontitis” and placed into two groups accordingly. The orally healthy group had no evidence of gingival inflammation, no periodontal pockets and no evidence of any other oral pathology. The “periodontitis” group had obvious gingival inflammation and periodontal pockets of over 5 mm in depth. No antimicrobial drugs had been given in the previous 8 weeks to any horse involved in the study.

### Sample collection

Once food debris was removed, an equine dental curette was used to collect subgingival plaque samples from a single periodontal pocket (depth greater than 5 mm) of 24 horses with clinical periodontitis and placed into 0.5 mL fastidious anaerobe broth (FAB). A swab of the gingival margin with sufficient pressure to also collect material from the gingival crevice on the buccal aspect of cheek teeth 307–308 (Modified Triadan Numbering System) was taken from 24 orally healthy horses using an Amies Transport Swab (VWR International, Lutterworth, UK). One periodontitis affected sample was lost for further sample processing, resulting in 23 samples from periodontitis cases and 24 samples from healthy horses being available for analysis. Post-mortem samples were collected within 1 hour of euthanasia.

### Sample processing and DNA extraction

Supragingival and subgingival plaque samples were each vortex mixed for 30 s and Amies transport swabs were immersed in 0.5 mL FAB and mixed to remove bacteria. A crude DNA extract was prepared from each sample by digestion with proteinase K (100 µg/mL) at 60 °C for 60 min, followed by boiling for 10 min. Further DNA purification was conducted using a bead beating technique where 150 µL of each sample was mixed with 200 µL phenol saturated with Tris–HCl (pH 8.0), 250 µL glass beads (0.1 mm) suspended in TE buffer and 200 µL lysis buffer. Samples were then placed in a BioSpec Mini-Beadbeater for 2 min at 2100 oscillations/min and DNA extracted with the AGOWA mag Mini DNA Isolation Kit (AGOWA, Berlin, Germany).

### High-throughput sequencing

For each sample, the V3–V4 region (which gives optimal taxonomic coverage and taxonomic resolution) of the bacterial 16S rRNA gene was generated by PCR with primers 341F (CCTACGGGNGGCWGCAG) and 806R (GGACTACHVGGGTWTCTAAT). Primers contained Illumina adapters and a unique 8-nucleotide sample index sequence key [15]. Amplicon libraries were pooled in equimolar amounts and purified using the Illustra™ GFXTM PCR DNA and Gel Band Purification Kit (GE Healthcare, Eindhoven, The Netherlands). Amplicon quality and size was analysed on an Agilent 2100 Bioanalyzer (Santa Clara, CA, USA). Paired-end sequencing of amplicons was conducted on the Illumina MiSeq platform using the v3 kit generating 2 × 301 nucleotide reads (Illumina, San Diego, USA).

### Analysis of sequencing data

Sequencing reads were merged [16], processed and clustered with USEARCH version 8.0.1623 [17]. After merging (minimum and maximum merged length, 380 and 438, respectively), the sequences were quality filtered (max. expected error rate 0.002, no ambiguous bases allowed) and clustered into operational taxonomic units (OTUs) using the following settings: -uparse_maxdball 1500, only de novo chimera checking, usearch_global with -maxaccepts 8 -maxrejects 64 -maxhits 1. QIIME version 1.8.0 [18] was used to select the most abundant sequence of each OTU and assigned a taxonomy using the RDP classifier [19] with a minimum confidence of 0.8 and the 97% representative sequence set based on the SILVA rRNA database, release 119 for QIIME [20]. Attributes such as oxygen utilisation, Gram stain and shape were assigned at genus level as previously described [21].

### Statistical analysis

In order to normalise the sequencing depth, the dataset was randomly sub-sampled to 16 000 reads per sample. Diversity analysis (Shannon Diversity Index, Chao-1 estimate of total species richness), data ordination by principal component analysis (PCA) and assessment of differences between microbial profiles of the two groups by one-way PERMANOVA were performed using PAleontological STatistics (PAST; v3.02) software [22]. PERMANOVA was used with Bray–Curtis similarity distance. For PCA, the OTU dataset was additionally normalized by log2-transformation. The difference in diversity of the genera detected in both health and disease was compared and analysed statistically using the Mann–Whitney U test in SPSS (version 21.0). To determine which OTUs and taxa contribute to differences between the groups, linear discriminant analysis effect size (LEfSe) [23] was used.

## Results

### Sample demographics

The majority (16 of 24; 66.7%) of the periodontitis samples originated from the Royal (Dick) School of Veterinary Studies, University of Edinburgh, three (12.5%) originated from the Weipers Centre Equine Hospital, University of Glasgow and five (20.8%) were post-mortem samples. The mean age of sampled horses with periodontitis was 13.2 years (range 3–27 years); 13 (54%) of these horses were mares and 11 (46%) were geldings. Of the 24 orally healthy horses sampled, 20 (83.3%) were collected at the Weipers Centre Equine Hospital, University of Glasgow, two (8.3%) at the Royal (Dick) School of Veterinary Studies, University of Edinburgh and two (8.3%) were post-mortem samples. The average age of this group was 11.7 years (range 4–27 years); 16 (66.7%) of horses were geldings and eight (33.3%) were mares. Of all mares included in the study, 52% had periodontitis and 40% of all geldings had periodontitis. There was however no statistically significant difference between healthy and periodontitis affected horses by gender (*p* = 0.383; Chi square test) or by age (*p* = 0.242; Mann–Whitney test).

A diverse range of breeds were sampled, although 19 of 48 (39.6%) were native ponies: Welsh Cob (*n* = 6), Welsh Pony (*n* = 4), Dartmoor Pony (*n* = 1), Shetland Pony (*n* = 2), Connemara Pony (*n* = 2), Exmoor Pony (*n* = 2), Highland Pony (*n* = 1), Fell Pony (*n* = 1). Eleven of 48 horses (22.9%) were Cobs or Cob crossbreeds and four horses (8.3%) were Thoroughbred (TB) or TB crossbreeds. Icelandic horses accounted for three (6.3%) of the samples. The remaining 11 (22.9%) horses were of a variety of breeds: Arabian (*n* = 3), Irish Sports Horse (*n* = 3), Gelderlander (*n* = 1), Trakehner (*n* = 1), Warmblood (*n* = 2), Irish Draft (*n* = 1). No significant difference was observed between breed and the presence of periodontitis.

### Sequencing output

Sequencing generated a total of 4 170 177 reads. After quality processing the OTU table contained 1 342 927 reads that were clustered in 1334 OTUs. The number of reads per sample ranged from 16 272 to 49 685 (median 27 855, mean 28 573, SD 7943). After subsampling at equal depth of 16 000 reads/sample, 1308 OTUs remained in the dataset that was used for the further analyses.

### Microbial profile analyses

**Figure 1:**
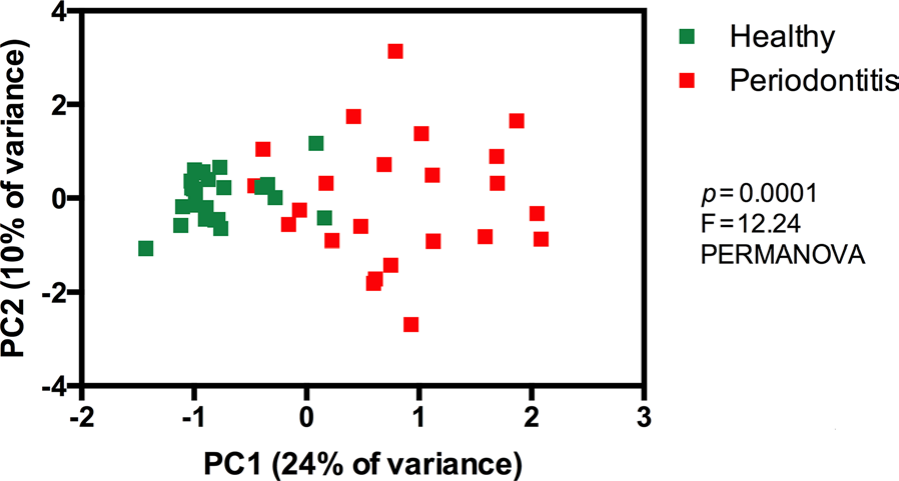
**Two-dimensional ordination of equine microbial profiles at oral health and periodontal disease by principal component analysis (PCA).** The OTU data was subsampled at 16 000 reads/sample and log2-transformed before the PCA.

**Figure 2:**
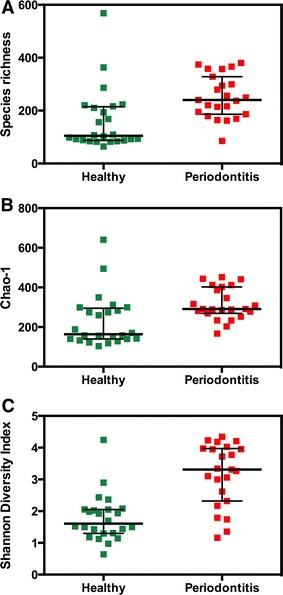
**Diversity analysis on equine microbial profiles at health and periodontal disease.** **A** Observed species richness or number of OTUs/sample; **B** estimated species richness or Chao-1 and **C** Shannon diversity index. Healthy microbiomes were statistically significantly less diverse than microbiomes with periodontal disease (*p* < 0.001, Mann–Whitney test).

Principal component analysis revealed clear differences between the equine oral microbiomes in oral health and periodontitis (Figure 1). Healthy samples clustered together and showed lower variability compared to periodontitis samples. The difference between microbial profiles of the two groups was statistically significant (*p* < 0.0001, F = 12.24, PERMANOVA). Microbial profiles from healthy horses were statistically significantly less diverse (*p* < 0.001, Mann–Whitney test), both by actual species richness (number of OTUs) (Figure 2A) as well as by estimated species richness or Chao-1 (Figure 2B) and Shannon Diversity Index (Figure 2C). On average, samples from healthy horses harboured 161 OTUs (SD 116, range 64–568), while samples from periodontitis affected horses contained 252 OTUs (SD 81, range 85–380).

### Compositional differences between the groups

Linear discriminant analysis (LDA) effect size (LEfSe) was used to assess the differences between the two groups of samples both at the OTU level and at the genus or higher taxonomic level. From all 1308 OTUs, 266 OTUs were statistically significantly different between the healthy and periodontitis groups (*p* < 0.05, LDA > 2). Of these, 64 OTUs had an absolute LDA score above 3 (Additional file 1), the majority of which (51 of 64 OTUs) were associated with disease.

**Figure 3:**
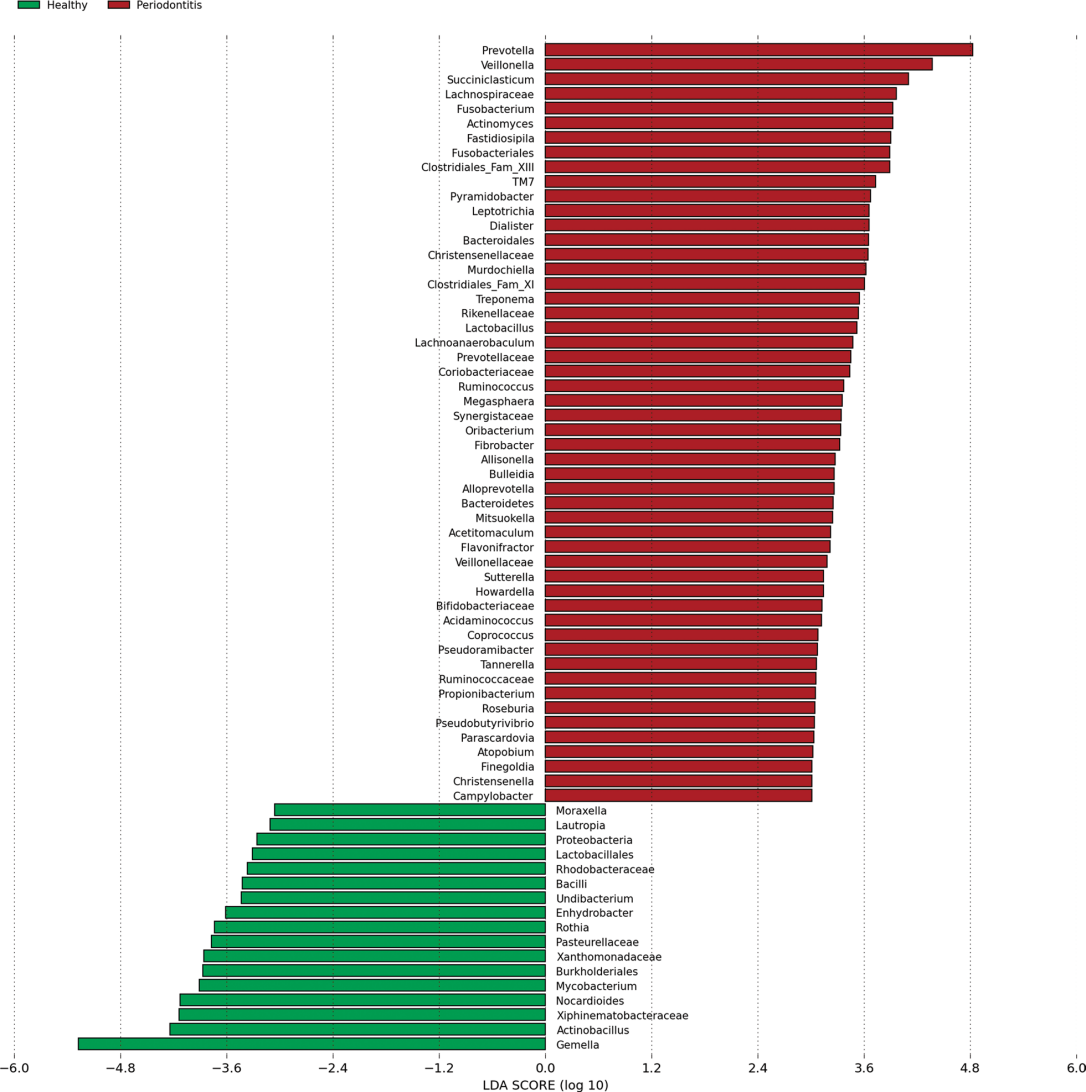
**Visualisation of most significant taxa (genus or higher level) that differentiate between health and periodontal disease in equine microbiomes.** In total 107 taxa were significantly different. Only 69 taxa that had LDA scores above 3 are shown. The taxa are ranked by the effect size in LEfSe.

**Figure 4:**
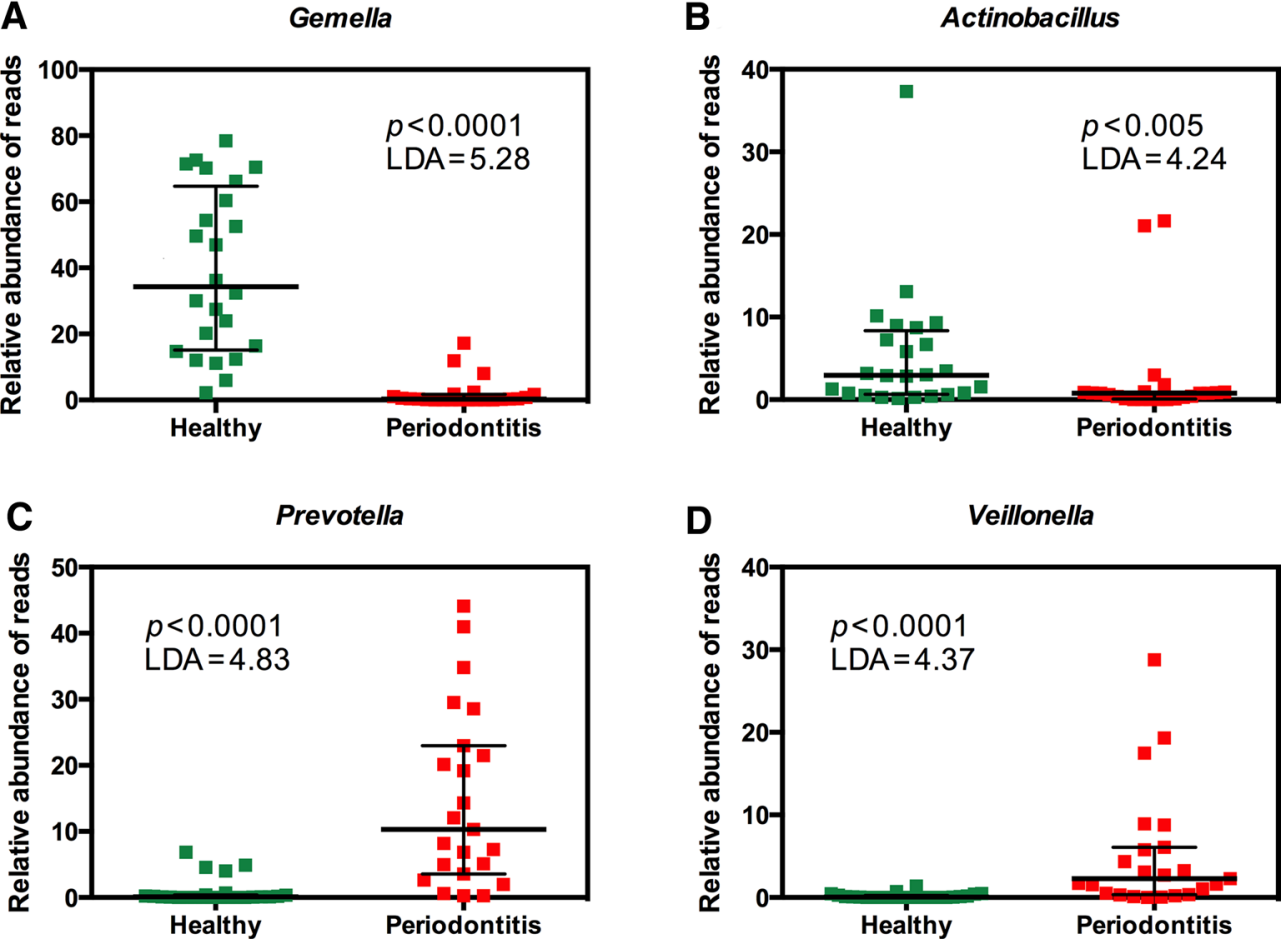
**The relative abundance of most discriminative genera between health and disease.** **A** *Gemella* and **B** *Actinobacillu*s in health; **C** *Prevotella* and **D** *Veillonella* in periodontitis, based on LDA scores in LEfSe. Values are expressed as a percentage.

At the genus level, from 356 genera or higher taxa, 107 taxa were statistically significantly different between the two groups (*p* < 0.05). Of these, 69 taxa had LDA scores above 3 and, again, the majority (52 of 69 taxa) were associated with disease (Figure 3). The most discriminative genera between health and disease were *Gemella* and *Actinobacillu*s in health and *Prevotella* and *Veillonella* in periodontitis, respectively (Figure 4).

**Figure 5:**
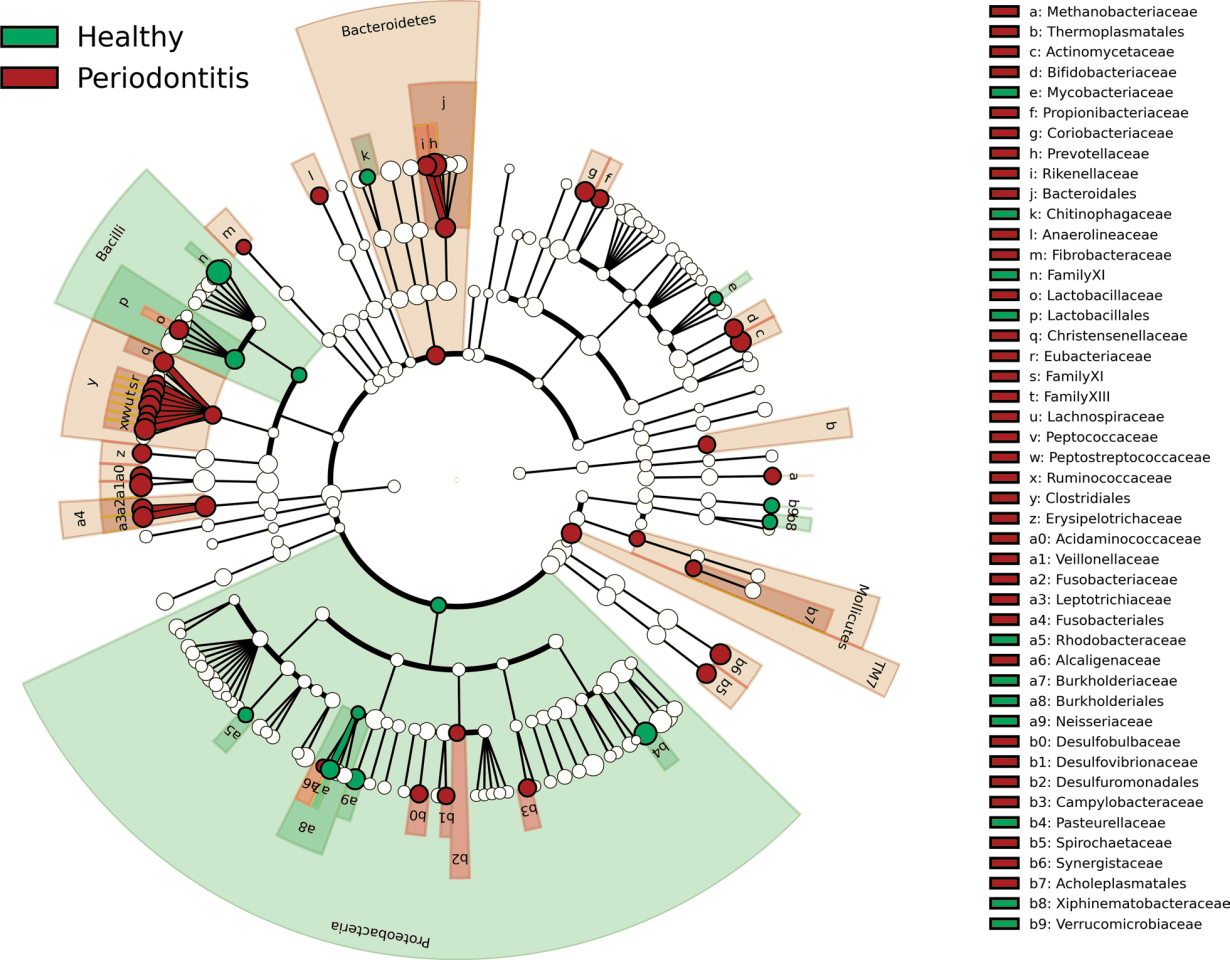
**Taxonomic representation of statistically significant differences between the healthy and periodontitis samples at family or higher taxonomic level.** All 51 significantly different taxa had LDA scores above 3. Differences are represented in colour (shades of red periodontal disease, green health).

From 179 entries at the family level, 51 were significantly different between health and disease (*p* < 0.05) (Figure 5). The majority (N = 38) of these were associated with disease, while only 13 microbial families were positively associated with health (Additional file 2). Interestingly, periodontitis samples had significantly higher relative abundance of Methanobacteriaceae (*p* = 0.0001) and Thermoplasmatales (*p* < 0.0001) (both families belong to the domain Archaea).

With regard to inferred Gram stain and shape, strongly significant differences were observed between healthy and diseased samples (**p < 0.0001, *p < 0.05, Mann–Whitney test; data not shown).

## Discussion

Despite the difficulty in permanently resolving equine periodontitis, its high prevalence and substantial effect on welfare, few original research studies on its aetiopathogenesis have been published. In humans, the disease is known to be multifactorial and although bacteria play a major role in the aetiopathogenesis of periodontitis in other species, their role in equine periodontitis has only recently received investigation [9]. Few studies have investigated the oral microbiome of the horse in oral health or disease. Recently, the microbiome of the equine gingival sulcus was investigated by pyrosequencing pooled samples from 200 sulcus sites in two orally healthy horses [24]. Twelve phyla were identified, the most prevalent being Gammaproteobacteria (28.8%), Firmicutes (27.6%) and Bacteroidetes (25.1%). The study suggested that there are many similarities between the equine subgingival microbiota and the subgingival microbiota detected in human, feline and canine studies. Putative periodontal pathogens such as *Treponema*, *Tannerella* and *Porphyromonas* species were detected at low levels in these samples. In addition, many bacteria identified were not closely related to other known bacteria and the authors suggested these may represent “equine-specific” taxa. As few previous studies have been performed investigating the equine oral microbiome, it is highly likely that novel, previously undetected bacteria will be identified when using modern, culture-independent techniques.

The current study was the first to use high-throughput 16S rRNA gene sequencing to compare the bacterial populations present in equine oral health and periodontitis and revealed a statistically significant dissimilarity between the bacterial populations found in equine oral health and in equine periodontitis lesions and represents a considerable advance on what has previously been documented for the oral microbial community in both healthy and diseased horses. In the current study, 60% of horses aged 10 years or above were affected by periodontitis and of all diseased horses, 70% were 10 years or older. Mares were found to be slightly more likely to have periodontitis than geldings (52% of mares compared to 40% of geldings), although this difference was not statistically significant. Due to the large variety of breeds sampled and the relatively small sample numbers, no particular breed disposition to disease could be identified. Further larger scale studies may be useful to examine links between equine periodontitis and age, sex and breed.

In this cross-sectional study it is impossible to equate the results with disease aetiology and pathogenesis. A potential limitation of this study is that samples were collected from both live and dead horses and that this could add further variability to the results. However, all samples were collected within 1 hour of death (usually much quicker) and, since DNA from live and dead bacteria was detected rather than live cells per se, it is very unlikely that any changes in the microbiomes would be attributable to death of the horses. In any case, individual healthy oral samples (whether from live or dead horses) demonstrated noticeable variation in the composition of their microbiomes but were more similar to each other than to those from horses with periodontitis, and vice versa. Longitudinal studies starting with young healthy horses, and follow-up on their periodontal status and microbiota of the oral cavity until development of periodontal disease would be required. The periodontal pocket found in diseased horses constitutes a new niche in an oral ecosystem that will select for a different microbiome and this may explain the significant increase in microbiome diversity noted in the periodontitis cases in comparison with the orally healthy horses. Increased microbiome diversity has also been noted in samples taken from human periodontitis patients in comparison to orally healthy controls [25, 26].

Environmental differences present between the healthy equine gingival sulcus and diseased periodontal pockets may be particularly striking in the horse, as equine dental anatomy allows for formation of particularly deep periodontal pockets which may measure over 15 mm in severe cases [9]. It is possible that during disease progression, the environmental changes occurring as a shallow gingival sulcus becomes a deep periodontal pocket allows a new group of bacteria to flourish whilst providing a less optimal environment for the growth of others. In the current study, significant differences were seen in both the expected shape and Gram staining characteristics of bacteria detected in oral health and periodontal pockets, with Gram negative rods, spirochetes and mycoplasma more evident in periodontitis.

Spirochetes have long been associated with human periodontitis [27] and more recently spirochetes were detected within the epithelium of equine periodontal pockets [9]. *Treponema denticola* is well recognised as a periodontal pathogen in man, acting as one of the three “red complex” bacteria found in severe periodontitis lesions alongside *Porphyromonas gingivalis* and *Tannerella forsythia* [28]. In another study, DNA corresponding to *Treponema* species was detected in 78.2% of horses with clinically overt equine odontoclastic tooth resorption hypercementosis (EOTRH) compared to 38% of unaffected horses and *Tannerella* DNA was found in 38.4% of diseased horses compared to 19% of unaffected horses [10]. In the current study, abundance of both the *Tannerella* and *Treponema* genera was significantly increased in periodontitis.

The most discriminative genera between health and disease were the genera *Gemella* and *Actinobacillus* in health and *Prevotella* and *Veillonella* in periodontitis, respectively. In equine periodontitis, the abundance of bacteria belonging to the *Prevotella* and *Veillonella* genera was significantly increased in comparison to oral health. Several species of *Prevotella* have been shown to be involved in human periodontitis, such as *Prevotella intermedia* and *Prevotellamelaninogenica* [29]. Several species of *Veillonella* have been isolated from both healthy gingival sulci and diseased periodontal pockets in man. However, *Veillonella parvula* has been significantly associated with chronic periodontitis [30]. Interestingly, *Prevotella intermedia* and *Prevotella nigrescens* have been shown to stimulate cytokine production by activation of Toll-like receptor 2 and *Veillonella parvula* has been shown to stimulate cytokine production by activation of both Toll-like receptor 2 and Toll-like receptor 4 [31]. This is of potential importance as the production of a destructive inflammatory response in periodontal tissue by stimulation of the innate immune system by periodontopathogenic bacteria is thought to be central in disease pathogenesis in man [32].

In equine oral health, significantly higher relative abundances of the genera *Gemella* (*p* < 0.0001) and *Actinobacillus* were noted in comparison to periodontitis, indicating that these genera comprise part of the normal oral flora of the horse. Bacteria belonging to the *Gemella* genus have been found to constitute high proportions of the microbiota of the dorsal surface of the human tongue [33]. In addition, *Actinobacillus equi* has been frequently isolated from the oral cavity of healthy horses [34, 35]. Given that no previous studies have characterised the equine oral microbiome in such detail, it is highly likely that many novel or previously uncharacterised bacteria are present in both oral health and periodontitis and additional studies would be required to further determine the composition of the equine oral microbiome.

In conclusion, the two cohorts of horses examined harboured highly distinct microbial profiles, with samples from periodontally affected horses being more diverse than samples from the healthy horses. Further, preferably longitudinal, studies are required to determine which bacteria are actively involved in the pathogenesis of disease.

## Additional files

10.1186/s13567-016-0333-1 **OTUs that differed significantly between the groups.** The output was created in LEfSe.
10.1186/s13567-016-0333-1 **Microbial families of higher taxa that differed significantly between the groups.** The output was created in LEfSe. The same taxa are visualised in Figure 5.

## Abbreviations

FAB: fastidious anaerobe broth
LDA: linear discriminant analysis
LEfSe: linear discriminant analysis effect size
OTU: operational taxonomic unit
PCA: principal component analysis
